# Correlation analysis of serum Orexin A, PBP4, and FGF19 levels with insulin resistance and neonatal weight in gestational diabetes mellitus: A cross-sectional study

**DOI:** 10.1097/MD.0000000000044040

**Published:** 2025-08-22

**Authors:** Xiaojie Jia

**Affiliations:** a Obstetrical Department, Shenzhen Futian District Maternal and Child Health Hospital, Shenzhen, China.

**Keywords:** BMI moderation, gestational diabetes mellitus, insulin resistance, neonatal weight

## Abstract

The purpose of this study was to investigate the relationships between serum biomarkers Orexin A, PBP4, FGF19, and insulin resistance as measured by HOMA-IR, and neonatal weight in patients with gestational diabetes mellitus (GDM). A total of 80 pregnant women diagnosed with GDM were enrolled in this cross-sectional study. Baseline characteristics, including age, body mass index (BMI), and gestational age, were recorded. Fasting blood samples were collected to assess serum levels of Orexin A, PBP4, and FGF19, as well as to calculate HOMA-IR as a measure of insulin resistance. Neonatal birth weight was documented at delivery. Statistical analyses included Pearson correlation, multivariate linear regression, and subgroup analysis based on median values of HOMA-IR and neonatal birth weight. A statistically significant positive correlation was observed between insulin resistance, and neonatal birth weight (*R* = 0.45, *P* < .001). The low HOMA-IR group has significantly higher levels of Orexin A, PBP4, and FGF19 compared to the high HOMA-IR group (*P*-values: .02, .04, and .01, respectively), indicating a negative association with insulin resistance. Orexin A, PBP4 and FGF19 are negatively associated with both insulin resistance (*P* < .01, *P* < .01, *P* = .03) and birth weight (*P* = .02, *P* < .01, *P* = .02). Furthermore, a moderation effect of BMI on the relationship between PBP4 levels and insulin resistance, with stronger associations observed in women with a higher BMI. The study reveals that serum Orexin A, PBP4, and FGF19 are significantly associated with insulin resistance and neonatal weight in GDM, with BMI modulating the impact of PBP4 on insulin resistance. These biomarkers could serve as predictive indicators for insulin resistance and fetal growth in GDM, suggesting potential mechanism of insulin resistance affecting newborn weight.

## 1. Introduction

Gestational diabetes mellitus (GDM), a form of diabetes that emerges during pregnancy, affects a significant proportion of pregnancies globally, with prevalence rates increasing globally, driven by factors such as maternal age, obesity, and genetic predisposition.^[1,2]^ GDM is linked to an array of adverse maternal and neonatal outcomes, such as preeclampsia, macrosomia, and the need for cesarean section, as well as long-term risks for both the mother and child, including Type II diabetes and obesity.^[3,4]^

Insulin resistance, a central feature of GDM, exhibits variability in severity among individuals. The homeostasis model assessment of insulin resistance (HOMA-IR) is a widely utilized method for assessing insulin resistance in both epidemiological studies and clinical settings, offering a reliable estimate of insulin sensitivity based on fasting glucose and insulin levels.^[5]^ Recent studies have highlighted the role of various serum biomarkers in the development of insulin resistance and related metabolic disorders. Orexin A, a neuropeptide involved in appetite and energy regulation, has been implicated in diabetes and obesity pathophysiology.^[6]^ Pre-beta lipoprotein 4 (PBP4), also known as apolipoprotein M, has been identified as a link between triglyceride-rich lipoprotein metabolism and insulin resistance.^[7]^ Fibroblast growth factor 19 (FGF19), or FGF15 in humans, plays a role in bile acid and glucose metabolism and has been associated with metabolic regulation and the development of insulin resistance.^[8]^ The impact of GDM on neonatal outcomes is well-documented, with macrosomia being a common complication. This can lead to difficulties during delivery and an increased risk of future metabolic disorders in the offspring.^[9,10]^ The role of maternal insulin resistance in fetal growth and development is complex and involves multiple hormonal and metabolic pathways.

Given the importance of insulin resistance in GDM and its potential influence on neonatal outcomes, there is a need to investigate the interplay between serum biomarkers, insulin resistance, and birth weight. This study aims to explore the correlation between serum levels of Orexin A, PBP4, and FGF19 with insulin resistance and neonatal weight in women with GDM, providing a comprehensive analysis of these relationships. Understanding these associations could provide valuable insights into the mechanisms underlying GDM and inform the development of novel therapeutic strategies.

## 2. Methods

### 2.1. Study design and participants

This cross-sectional study was conducted at our hospital from January 2022 to January 2024. We enrolled 80 pregnant women diagnosed with GDM according to the American Diabetes Association criteria (2018). Specifically, GDM was diagnosed using a one-step 75-g oral glucose tolerance test with the following diagnostic thresholds: fasting plasma glucose ≥ 5.1 mmol/L, 1-hour glucose ≥ 10.0 mmol/L, or 2-hour glucose ≥ 8.5 mmol/L. Meeting any one of these criteria was sufficient for a diagnosis of GDM. The inclusion criteria were: women aged 18 to 40 years, diagnosed with GDM between the 24th and 28th weeks of gestation. Exclusion criteria included preexisting diabetes, multiple pregnancies, and chronic inflammatory or endocrine disorders. The study was approved by the Institutional Review Board of the Maternity and Child Health-care Hospital of Futian District (Approval No. KY-2024015), and informed consent was obtained from all participants.

### 2.2. Clinical information collection

Maternal anthropometric measurements, including weight and height, were recorded to calculate body mass index (BMI). Gestational age was confirmed through an ultrasound scan. Blood samples were collected after an overnight fast to measure fasting plasma glucose, fasting insulin, and the specific biomarkers of interest (Orexin A, PBP4, and FGF19). Fasting insulin levels were measured using a chemiluminescent immunoassay analyzer and were used to calculate HOMA-IR as a measure of insulin resistance. Newborn birth weight was recorded at delivery.

### 2.3. Insulin resistance assessment

Insulin resistance was assessed using the Homeostasis Model Assessment of Insulin Resistance (HOMA-IR) formula: HOMA-IR = fasting insulin (μU/mL) × fasting glucose (mmol/L)/22.5. Fasting insulin and glucose levels were measured using a chemiluminescent immunoassay analyzer.

### 2.4. Measurement of serum biomarkers

Blood samples were centrifuged at 3000 rpm for 10 minutes to obtain serum, which was subsequently stored at −80°C until analysis. Levels of Orexin A, PBP4, and FGF19 were measured using commercially available enzyme-linked immunosorbent assay (ELISA) kits following the manufacturer’s instructions. The specific ELISA kits used were as follows: Orexin A: Human Orexin A ELISA Kit, Catalog No. E-EL-H0673, Elabscience Biotechnology Inc., Wuhan, China; PBP4 (Apolipoprotein M): Human Apolipoprotein M (APOM) ELISA Kit, Catalog No. E-EL-H1547, Elabscience Biotechnology Inc.; FGF19: Human FGF19 ELISA Kit, Catalog No. E-EL-H6158, Elabscience Biotechnology Inc. All samples were assayed in duplicate, and the intra-assay and inter-assay coefficients of variation were <8% and <10%, respectively.

### 2.5. Statistical analysis

Statistical analyses were performed using SPSS software version 26.0 (IBM Corp., Armonk). Data were expressed as mean ± standard deviation (SD) or median (interquartile range) as appropriate. Comparisons between groups were made using independent *t*-tests or Mann–Whitney *U* tests for continuous variables and χ^2^ tests for categorical variables. Pearson correlation coefficients were calculated to assess the associations between serum biomarker levels (Orexin A, PBP4, FGF19) and clinical outcomes like HOMA-IR and neonatal birth weight. Multivariate linear regression models were applied to evaluate the independent association of each biomarker with insulin resistance and birth weight. We adjusted for age, BMI, and gestational age as potential confounders, given their known associations with insulin resistance and fetal growth. Other variables considered as potential confounders included family history of diabetes, parity, and physical activity level. However, these variables were not included in the final models because preliminary analyses showed that they did not significantly alter the main associations and were not statistically significant predictors in the models. Statistical significance was set at a 2-sided *P*-value of <.05. Participants were stratified into groups based on the median values of HOMA-IR and neonatal birth weight. The median was chosen as the cutoff point to ensure balanced group sizes and to facilitate exploratory comparisons between higher and lower levels of insulin resistance and birth weight. Interaction effects between BMI and serum PBP4 levels on the outcomes were evaluated using moderated regression analysis.

## 3. Results

### 3.1. Baseline characteristics of participants

The baseline demographic and clinical characteristics are presented in Table 1. The mean age was 32.5 ± 4.2 years, with a median gestational age of 28.4 weeks (IQR 27–29 weeks). The mean BMI was 28.7 ± 3.8 kg/m^2^, and the average fasting plasma glucose was 5.8 ± 0.9 mmol/L. Neonatal birth weight averaged 3400 ± 450 grams.

**Table 1:** Baseline characteristics of study participants.

Parameter	Total (n = 80)
Age (yr)	32.5 ± 4.2
Gestational age (wk)	28.4 (27–29)
BMI (kg/m^2^)	28.7 ± 3.8
Fasting plasma glucose (mmol/L)	5.8 ± 0.9
Neonatal birth weight (g)	3400 ± 450

### 3.2. Correlation between insulin resistance and neonatal birth weight

A statistically significant positive correlation was observed between insulin resistance, measured via HOMA-IR, and neonatal birth weight (*R* = 0.45, *P* < .001). Notably, women in the higher HOMA-IR group (above median) exhibited significantly elevated birth weights compared to those in the lower HOMA-IR group (*P* = .02).

### 3.3. Biomarker levels across insulin resistance and birth weight groups

Orexin A, PBP4 and FGF19 levels are significantly higher in the low HOMA-IR group compared to the high HOMA-IR group, with *P*-values of 0.02, 0.04, and 0.01, respectively, indicating a negative association with insulin resistance. Similarly, these biomarkers show a trend or significant decrease in the higher birth weight group, with *P*-values of .08 for Orexin A, .01 for PBP4 and FGF19 (Table 2).

**Table 2:** Serum biomarker levels by HOMA-IR and birth weight groups.

Biomarker	Low HOMA-IR group	High HOMA-IR group	*P*	Low birth weight group	High birth weight group	*P*
Orexin A (pg/mL)	26.0 ± 6.5	21.0 ± 7.0	.02	25.5 ± 6.8	22.5 ± 6.9	.08
PBP4 (ng/mL)	88.5 ± 14.0	82.0 ± 15.9	.04	87.0 ± 13.8	83.5 ± 15.6	.01
FGF19 (pg/mL)	105.0 ± 21.0	90.0 ± 23.4	.01	103.0 ± 22.5	92.0 ± 23.0	.01

FGF19 = fibroblast growth factor 19, HOMA-IR = homeostasis model assessment of insulin resistance, PBP4 = pre-beta lipoprotein 4.

### 3.4. Association of biomarkers with insulin resistance and birth weight

Table 3 demonstrates that Orexin A, PBP4 and FGF19 are negatively associated with both insulin resistance (*P* < .01, *P* < .01, *P* = .03) and birth weight (*P* = .02, *P* < .01, *P* = .02). For every one-unit increase in Orexin A levels, insulin resistance (HOMA-IR) is estimated to decrease by 0.38 units (95% CI: −0.55 to −0.21, *P* < .01). Similarly, neonatal birth weight is estimated to decrease by 0.30 grams (95% CI: −0.45 to −0.15, *P* = .02) for each one-unit increase in Orexin A levels, holding other variables constant. For every one-unit increase in PBP4 levels, insulin resistance (HOMA-IR) is estimated to decrease by 0.50 units (95% CI: −0.65 to −0.35, *P* < .01). Neonatal birth weight is also estimated to decrease by 0.35 grams (95% CI: −0.50 to −0.20, *P* < .01) for each one-unit increase in PBP4 levels, after adjusting for other variables. For every one-unit increase in FGF19 levels, insulin resistance (HOMA-IR) is estimated to decrease by 0.20 units (95% CI: −0.35 to −0.05, *P* = .03). Neonatal birth weight is estimated to decrease by 0.28 grams (95% CI: −0.43 to −0.13, *P* = .02) for each one-unit increase in FGF19 levels, holding other variables constant. These findings suggest that these biomarkers may play a role in the pathophysiology of insulin resistance and fetal growth in GDM.

**Table 3:** Multivariate associations of biomarkers with HOMA-IR and birth weight.

Biomarker	HOMA-IR		Birth weight	
	β (95% CI)	*P*	β (95% CI)	*P*
Orexin A	−0.38 (−0.55 to −0.21)	<.01	−0.30 (−0.45 to −0.15)	.02
PBP4	−0.50 (−0.65 to −0.35)	<.01	−0.35 (−0.50 to −0.20)	<.01
FGF19	−0.20 (−0.35 to −0.05)	.03	−0.28 (−0.43 to −0.13)	.02

FGF19 = fibroblast growth factor 19, HOMA-IR = homeostasis model assessment of insulin resistance, PBP4 = pre-beta lipoprotein 4.

### 3.5. BMI moderation of PBP4 and insulin resistance

Moderation analysis illustrated a significant interaction effect of BMI on the relationship between PBP4 levels and insulin resistance. In women with a BMI greater than 30 kg/m^2^, elevated PBP4 levels were associated with a greater increase in HOMA-IR (β = 0.68, *P* < .001). Conversely, among participants with a BMI less than 30 kg/m^2^, the association was notably weaker (β = 0.15, *P* = .35).

## 4. Discussion

These findings suggest a potential involvement of these markers in both insulin resistance and fetal growth regulation. BMI modifies the relationship between PBP4 and insulin resistance, with stronger effects observed in participants with a BMI > 30 kg/m^2^. The findings contribute to the existing body of literature by providing novel insights into the potential roles of these biomarkers in the context of GDM.

Our results indicate a negative correlation between Orexin A levels and insulin resistance in GDM patients, which is consistent with previous research suggesting that Orexin A may be implicated in glucose metabolism and energy homeostasis.^[11,12]^ The exact mechanisms through which Orexin A influences insulin sensitivity remain to be fully elucidated; however, it is hypothesized that its role in regulating appetite and energy expenditure may contribute to the improvement of insulin resistance.^[13]^

The strong association between PBP4 levels and insulin resistance observed in our study is in line with recent findings that have identified PBP4 as a potential biomarker for evaluating insulin resistance and β-cell dysfunction.^[14]^ PBP4, also known as apolipoprotein M, has been shown to strengthen insulin signaling, potentially contributing to the improvement of insulin resistance.^[15]^ As an apolipoprotein, PBP4 may enhance insulin sensitivity by facilitating blood lipid metabolism and expediting glucose uptake, thereby delaying to elevated neonatal birth weight.^[16]^

FGF19, a hormone involved in bile acid and glucose metabolism, has been reported to have insulin-sensitizing effects, which may protect against the development of insulin resistance.^[17]^ Study shows that FGF19 interacts with the FXR nuclear receptor to regulate bile acid synthesis and glucose metabolism, which can reduce hepatic glucose output and improve insulin sensitivity, mitigating the risks associated with GDM.^[18]^

The associations between the biomarkers and neonatal weight observed in our study suggest that these factors may play a role in fetal growth and development. The negative association between Orexin A and neonatal weight may reflect the influence of maternal appetite regulation on fetal growth, while the negative association with FGF19 could indicate a protective role against excessive fetal growth.^[19]^ The moderation analysis revealed a significant interaction effect of BMI on the relationship between PBP4 levels and insulin resistance. This finding suggests that the impact of PBP4 on insulin resistance may be more pronounced in women with a higher BMI. This is consistent with Xu’s findings,^[20]^ which show that PBP4 levels are higher in obese patients because they have lower leptin and higher cholesterol. It highlighting the potential importance of BMI in the management of GDM.

Orexin A and PBP4 could serve as early biomarkers for heightened insulin resistance and macrosomia risk in GDM pregnancies, enabling more targeted interventions. FGF19 might represent a therapeutic target for reducing metabolic risks in GDM. Given the interaction between BMI and PBP4, clinicians could consider BMI stratification when evaluating metabolic risks and planning interventions.

The current study offers valuable insights into the associations among serum biomarkers, insulin resistance, and neonatal weight in patients with GDM; however, certain limitations should be acknowledged. Firstly, the study’s cross-sectional design limits causal inferences. A larger cohort and longitudinal design would enhance the robustness of findings. Secondly, variables such as dietary intake, physical activity, and genetic predispositions were not accounted for, which could influence results.

Lastly, the study did not capture temporal changes in biomarker levels across different gestational stages. Additionally, the sample size was relatively small, and a formal power analysis was not conducted prior to data collection. Consequently, there is a risk of Type II errors, meaning that some true associations between biomarkers and outcomes may not have been detected. Future studies with larger sample sizes and formal power calculations are needed to confirm our findings and reduce the risk of Type II errors.

## 5. Conclusion

In conclusion, our study provides evidence for the involvement of Orexin A, PBP4, and FGF19 in the pathophysiology of insulin resistance and neonatal weight in GDM. These biomarkers could serve as potential therapeutic targets or diagnostic tools in the future management of GDM.

## Acknowledgments

We greatly appreciate all of the study participants.

## Author contributions

**Conceptualization:** Xiaojie Jia.

**Data curation:** Xiaojie Jia.

**Investigation:** Xiaojie Jia.

**Methodology:** Xiaojie Jia.

**Validation:** Xiaojie Jia.

**Visualization:** Xiaojie Jia.

**Writing – original draft:** Xiaojie Jia.

**Writing – review & editing:** Xiaojie Jia.
